# Brain organoids as precision models for neurodegenerative diseases: from disease modeling to drug discovery

**DOI:** 10.3389/fnins.2026.1764964

**Published:** 2026-02-18

**Authors:** Yanxu Zheng, Wenke Zhou, Haozhe Chang, Kuihong Zheng

**Affiliations:** 1 Department of Radiology, The Sixth Medical Center of PLA General Hospital, Beijing, China; 2 Xiangya School of Medicine, Central South University, Changsha, Hunan, China

**Keywords:** brain organoids, CRISPR-Cas9 genome editing, drug discovery, induced pluripotent stem cells (iPSCs), neurodegenerative diseases

## Abstract

Neurodegenerative diseases such as Alzheimer’s disease (AD), Parkinson’s disease (PD), and amyotrophic lateral sclerosis (ALS) have become major global causes of disability and mortality. Their complex pathogenic mechanisms remain incompletely understood, and effective disease-modifying therapies are still lacking. Traditional animal models and two-dimensional (2D) cell culture systems exhibit notable limitations in structural complexity, human relevance, and translational validity, making it difficult to faithfully recapitulate human-specific neuropathology. In recent years, brain organoid technology derived from induced pluripotent stem cells (iPSCs) has advanced rapidly, enabling the self-organization of diverse neuronal and glial cell types within a three-dimensional (3D) architecture that partially mimics human brain development and disease-related pathological events. When integrated with CRISPR–Cas9-based genome editing and multi-omics profiling, organoids support causal mechanism studies, target validation, and individualized drug-response prediction, highlighting their growing value in early-stage drug discovery. Despite current challenges—including insufficient maturation, lack of vascularization and immune components, and batch variability—the continuous progress in bioengineering, microfluidic systems, and artificial intelligence (AI)–driven multimodal data analysis is steadily expanding the translational potential of organoids as human-relevant preclinical models. Overall, brain organoids provide an essential foundation for constructing physiologically relevant and predictive research platforms for neurodegenerative diseases, offering new opportunities for therapeutic development and precision medicine.

## 1 Introduction

Neurodegenerative diseases such as Alzheimer’s disease (AD), Parkinson’s disease (PD), and amyotrophic lateral sclerosis (ALS) represent a major and growing public health challenge worldwide. With rapid population aging, their prevalence continues to increase, leading to progressive and irreversible damage to the nervous system and becoming one of the leading causes of disability and mortality (GBD 2021 Neurology Collaborators, 2022). Although substantial advances have been made in elucidating disease mechanisms—including amyloid-*β* (Aβ) deposition, *α*-synuclein (α-syn) aggregation, and TAR DNA-binding protein 43 (TDP-43) mislocalization—most neurodegenerative disorders still lack effective therapies capable of halting or reversing disease progression (Karran and De Strooper, 2022).

Traditional research models, including rodent models and 2D cell culture systems, have long served as foundational tools for studying neurodegeneration and drug development, yet their limitations have become increasingly evident. Species differences prevent animal models from faithfully recapitulating human-specific brain architecture and pathological trajectories, limiting the translational predictive value of preclinical findings. Likewise, 2D cultures lack the 3D cell–cell and cell–matrix interactions necessary to reconstruct the complex human neural microenvironment, making them insufficient for modeling synaptic connectivity, circuit maturation, and multicellular pathological processes (Qian et al., 2020; Papaspyropoulos et al., 2020). These shortcomings are a key factor in the high attrition rate of drug candidates for neurodegenerative diseases in clinical trials. For instance, a systemic review indicates that between 2004 and 2021, approximately 98 AD drug candidates failed in Phase II/III trials while only 2 succeeded, representing a success rate of only about 2% (Kim et al., 2022). Similarly, a meta-analysis of amyotrophic lateral sclerosis trials reported an overall attrition rate of 32%, which increased significantly with longer trial duration (van Eijk et al., 2024). This persistent challenge underscores the urgent need for more human-relevant and physiologically accurate modeling systems.

In recent years, brain organoid technology derived from human iPSCs has emerged as a transformative approach for studying neurodegeneration. Brain organoids can self-organize into 3D structures that recapitulate key aspects of human brain development, including cellular diversity and region-specific organization, and can mimic essential pathological events such as Aβ production, mcrotubule-associated protein tau (Tau) hyperphosphorylation, *α*-syn accumulation, and motor neuron (MN) degeneration (Pască, 2022). Importantly, organoids retain the donor’s genetic background and can be paired with clustered regularly interspaced short palindromic repeats (CRISPR)- CRISPR associated protein 9 (Cas9)-based genome editing to create isogenic controls, enabling precise disease modeling, mechanistic dissection, target validation, and individualized drug-response prediction (Velasco et al., 2020). Owing to these advantages, brain organoids are becoming a central tool in neurodegenerative disease research and therapeutic development.

This review therefore provides a systematic overview of recent advances in organoid-based modeling of AD, PD, and ALS, highlighting the unique strengths of organoids in recapitulating key pathological events. We further summarize emerging evidence on organoid applications in drug discovery, pharmacological assessment, and target validation, illustrating their value in addressing pathological processes that traditional models fail to capture. Finally, we discuss current challenges—including insufficient maturation, lack of immune and vascular components, and batch variability—and consider future directions informed by bioengineering innovations, multi-omics integration, and AI-driven modeling. Through this synthesis, we aim to provide a conceptual and methodological framework for advancing human-relevant neurodegenerative disease research and accelerating precision drug development.

## 2 Advantages and principles of brain organoids as precision models

The emergence of human iPSCs has laid the foundation for constructing individualized neural models, enabling researchers to recapitulate human neurodevelopment *in vitro* under patient-specific genetic backgrounds. This capability provides a critical opportunity to dissect the genetic heterogeneity underlying neurodegenerative diseases (Yamanaka, 2020). The process of creating brain organoids involves reprogramming various somatic cells into iPSCs, which can either spontaneously differentiate into heterogeneous brain-like tissues or be guided to form specific tissue types or “assembloids” that simulate interactions among various brain regions (Kadoshima et al., 2013; Xiang et al., 2021; Bagley et al., 2017). Notably, pioneering work by Kadoshima et al. (2013) in establishing self-organizing three-dimensional neural cultures provided a seminal foundation for the development of modern brain organoid protocols. Building on this, organoid technology has rapidly evolved. Early studies relied primarily on 2D neural induction systems, which were inherently limited in their ability to reproduce the spatial architecture and cellular diversity of the human brain. Since the pioneering work of Lancaster and Knoblich, who generated self-organizing cerebral organoids with layered cortical structures, 3D brain-like models have increasingly become indispensable tools for probing complex neural processes (Lancaster et al., 2013; Lancaster and Knoblich, 2014). The development of region-specific organoids—including cortical, midbrain, hypothalamic, and spinal cord organoids—has further expanded the utility of this technology, allowing researchers to model regionally selective pathologies in disorders such as AD, PD, and ALS (Qian et al., 2016; Jo et al., 2016).

A central advantage of organoid technology lies in its capacity for self-organization within a 3D microenvironment, giving rise to diverse cell types that closely follow human neurodevelopmental trajectories. These include excitatory and inhibitory neurons, astrocytes, and oligodendrocyte-lineage cells, among other (Di Lullo and Kriegstein, 2017; Gabriel and Gopalakrishnan, 2017). Within organoids, these cells engage in highly dynamic interactions, enabling the study of synaptogenesis, myelination, neuroinflammation, and early neural network activity with a degree of physiological relevance unattainable in traditional models (Qian et al., 2020). Given the fundamental species differences that limit the translational relevance of animal models, and the inability of 2D cultures to reconstruct 3D tissue architecture, organoids offer clear advantages in recapitulating complex, human-specific disease processes.

At the same time, the integration of CRISPR–Cas9 genome engineering has greatly enhanced the precision of organoid-based mechanistic studies. By generating isogenic pairs—edited and unedited lines within the same genetic background—researchers can systematically evaluate the functional impact of pathogenic mutations while minimizing confounding effects inherent to patient–control comparisons (Meng et al., 2025). Furthermore, combining organoids with single-cell transcriptomics, chromatin accessibility profiling, proteomics, and metabolomics enables high-resolution mapping of disease progression at the cellular level. These multimodal analyses help uncover alterations in cell fate trajectories, inflammatory networks, synaptic function, and metabolic pathways (Velasco et al., 2020; Jo et al., 2016). Together, these capabilities have transformed organoids from simple structural models into comprehensive platforms for mechanistic investigation, target validation, and drug action profiling.

**Figure 1:**
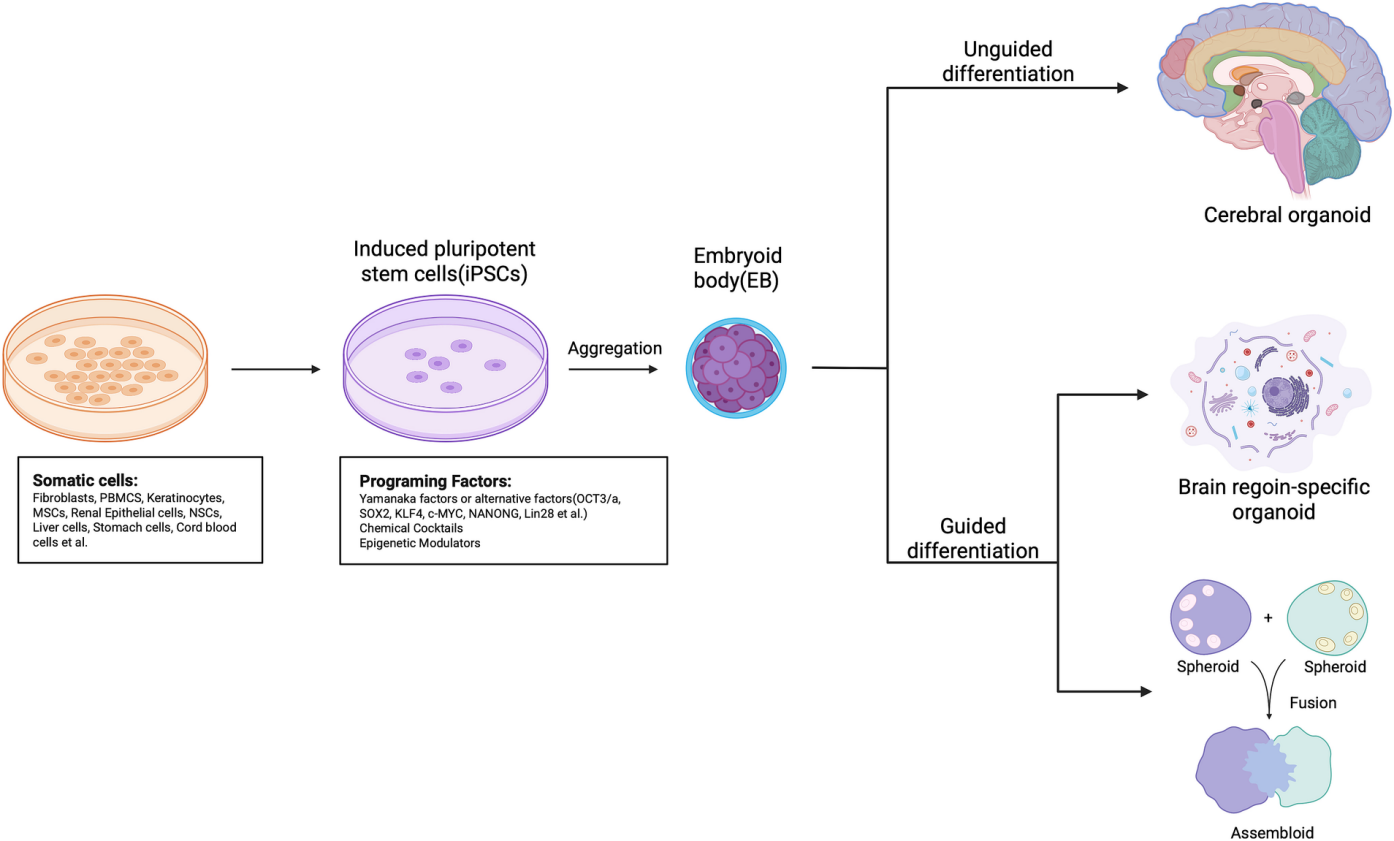
The process of creating brain organoids. Various somatic cell sources, including fibroblasts, PBMCs, and NSCs, can be reprogrammed *in vitro* into induced pluripotent stem cells (iPSCs) using methods such as OSKM or chemical cocktails. Unguided iPSCs (top) utilize the inherent signaling and self-organizing abilities of human pluripotent stem cells (hPSCs) to spontaneously differentiate into tissues that resemble the developing brain. These cerebral organoids typically consist of heterogeneous tissues that mimic different brain regions. In contrast, guided approaches (bottom) deploy small molecules and growth factors to create spheroids that represent a specific tissue type. To create brain region-specific organoids, patterning factors are applied early on to direct progenitor fate, and these factors are subsequently removed during later differentiation stages. Moreover, guided techniques can produce multiple spheroids or organoids that represent distinct brain region identities, which can then be fused to create “assembloids” that simulate interactions among various brain regions.

Overall, the convergence of human cellular origin, 3D architecture, developmental dynamics, self-organizing capacity, and compatibility with genome editing and multi-omics technologies positions brain organoids as highly promising precision models for neurodegenerative disease research. These attributes offer substantial opportunities for advancing therapeutic discovery and enabling personalized medicine (Figure 1).

## 3 Advances in organoid-based modeling of major neurodegenerative diseases and their pharmacological implications

### 3.1 Alzheimer’s disease (AD) organoid models

AD, the most prevalent neurodegenerative disorder, is characterized by hallmark pathological features including Aβ deposition, Tau hyperphosphorylation, synaptic dysfunction, and neuroinflammation (Scheltens et al., 2021). Traditional animal models fail to simultaneously recapitulate these events and cannot reproduce human-specific characteristics such as the Aβ42/Aβ40 ratio or Tau aggregation patterns (Drummond and Wisniewski, 2017). iPSC-derived AD organoids have achieved notable progress in reconstructing key molecular pathologies, providing a human-relevant system for mechanistic investigation.

Early studies demonstrated that organoids carrying familial AD mutations (e.g., APP, Presenilin1, Presenilin2) spontaneously developed measurable Aβ accumulation, Tau hyperphosphorylation, and neuronal injury—closely resembling pathological cascades observed in patient brains (Choi et al., 2014). These findings provided a valuable *in vitro* model that recapitulates both Aβ accumulation and Tau hyperphosphorylation from an endogenous source, offering a platform to study their potential interaction without external induction. Subsequent work extended culture duration, enhanced neuronal maturation, and incorporated astrocytes, yielding additional phenotypes linked to disease progression, including reduced synaptic density and impaired neuronal network activity (Raja et al., 2016). Multicellular interaction models further enabled the recapitulation of more complex features such as glial activation, Tau propagation, and metabolic reprogramming, with some systems even exhibiting early neurofibrillary tangle–like structures (Cerneckis et al., 2023; Gonzalez et al., 2018).

AD organoids have also become valuable tools for drug discovery and pharmacological validation. *γ*-Secretase modulators, *β*-secretase inhibitors, and Tau-targeting molecules or antibodies have been shown to reduce Aβ production, lower phosphorylated Tau (p-Tau) levels, and restore synaptic markers within organoids (Park et al., 2021). Because organoids retain patient-specific genetic backgrounds, such assays can more accurately capture individualized drug responses and inform precision therapeutic strategies. With improved scalability, organoid platforms now support multiplexed readouts—such as neural network activity, inflammatory states, and metabolic function—broadening their utility in phenotypic screening (Sharifi et al., 2024).

The integration of CRISPR–Cas9 editing with organoid systems further enables causal pathway validation (Cerneckis et al., 2023). For example, knockout of *β*-site APP-cleaving enzyme (BACE) 1 or modulation of sortilin-related receptor 1 (SORL1) in isogenic backgrounds allows direct assessment of their roles in Aβ accumulation and Tau pathology, confirming their relevance as therapeutic targets (Zhang et al., 2024). AD organoids have also opened new avenues for studying apolipoprotein E (ApoE) isoform–specific effects, microglia–neuron interactions, and lipid droplet dysregulation (Kim et al., 2024).

Overall, AD organoids enhance the predictive power of *in vitro* drug testing by reconstructing key molecular and synaptic pathologies, and they provide a robust platform for identifying and validating therapeutic targets.

### 3.2 Parkinson’s disease (PD) organoid models

PD is defined by progressive loss of dopaminergic (DA) neurons in the substantia nigra pars compacta and pathological aggregation of *α*-syn, accompanied by lysosomal–autophagic impairment and mitochondrial dysfunction (Dickson, 2018). Recent advances in human midbrain organoids (MOs) have demonstrated strong capability in modeling these pathological processes.

Rosety et al. (2023) reported that patient-derived organoids carrying Glucosidase Beta Acid (GBA) 1 mutations exhibited reduced glucocerebrosidase (GCase) activity, lipid metabolism disruption, autophagic blockade, and impaired DA neuron maturation—consistent with early GBA-PD pathology (Cerneckis et al., 2023). These organoids also spontaneously accumulated *α*-syn oligomers and displayed lysosomal dysfunction–associated cytotoxicity, underscoring their value for modeling *α*-synucleinopathy (Kim M. S. et al., 2023). MOs carrying Synuclein Alpha triplication further demonstrated gene dosage effects, with elevated *α*-syn levels, increased DA neuron vulnerability, and insufficient lysosomal acidification—closely aligning with clinical PD mechanisms (Cui et al., 2024). Krüger et al. (2024) additionally found strong associations between α-syn aggregation and astrocyte senescence markers, highlighting the ability of MOs to model critical neuron–glia interactions in PD. Metabolic and energetic phenotypes in PD organoids have also gained attention. Giorgi et al. noted that MOs exhibit oxidative-stress sensitivity and mitochondrial dependence similar to human substantia nigra neurons, supporting their use in exploring metabolic vulnerability (Giorgi et al., 2024). Ferroptosis-related lipid peroxidation signatures were also observed, suggesting that organoids effectively capture metabolic dysfunction in PD (Giorgi et al., 2024).

Building on their robust disease modeling capacity, midbrain organoids have been directly employed in pharmacological research and candidate drug evaluation for PD. For instance, in patient-derived organoids carrying GBA1 mutations, the investigational drugs ambroxol and GZ667161—both GBA pathway modulators—were demonstrated to effectively reduce pathological *α*-syn accumulation, validating the utility of this platform for preclinical screening (Kim M. S. et al., 2023; Zagare et al., 2022). Furthermore, single-cell RNA sequencing of LRRK2-G2019S-mutant midbrain organoids has recapitulated disease-associated transcriptomic signatures and altered developmental trajectories, providing a molecular benchmark for assessing drug-induced normalization (Zagare et al., 2022). Complementing these molecular readouts, an integrated 3D microelectrode array platform has enabled real-time monitoring of network-level electrophysiological activity in organoids, offering a sensitive functional outcome measure for evaluating compounds such as LRRK2 inhibitors (An et al., 2024; Elvira et al., 2025; Martinelli et al., 2024). These integrated approaches demonstrate that midbrain organoids serve as a multifaceted preclinical platform, bridging molecular mechanisms, cellular pathology, and functional network outcomes to significantly de-risk therapeutic development for Parkinson’s disease.

Taken together, midbrain organoids can robustly model DA neuron vulnerability, *α*-syn aggregation, and lysosomal/mitochondrial dysfunction while supporting mechanistic discovery and drug screening. Their human relevance and scalability position them as promising tools for PD preclinical research.

### 3.3 ALS and motor neuron disease organoid models

ALS involves progressive degeneration of upper and lower Motor Neurons (MNs), driven by disruptions in RNA metabolism, proteostasis, synaptic signaling, axonal transport, and non–cell-autonomous toxicity (Bonafede and Mariotti, 2017). Although iPSC-derived 2D systems and transgenic animals have clarified mechanisms of SOD1, TDP-43, Fused in Sarcoma (FUS), and chromosome 9 open reading frame 72 (C9ORF72), they cannot reconstruct multicellular interactions and hierarchical pathology within the human nervous system (Ma et al., 2025; Balusu et al., 2023). Organoid technologies now offer a more physiologically relevant means of modeling ALS pathology across molecular and cellular scales.

Cortical organoids derived from ALS patient iPSCs show TDP-43 hyperphosphorylation and aggregation, along with prion-like propagation through neuronal networks, accompanied by glial activation, DNA damage, and programmed cell death—all consistent with human pathology (Tamaki et al., 2023). C9ORF72 expansion organoids recapitulate nuclear RNA foci, dipeptide repeat (DPR) accumulation, and suppression of DNA repair pathways, indicating that organoids capture multilayered cellular stress responses (van der Geest et al., 2024). Mitochondrial profiling further shows increased mitochondrial DNA (mtDNA) mutations in C9ORF72 astrocytes, correlating with energetic deficits (Nie et al., 2025).

Spinal organoids and organoid-on-chip systems complement these models by reconstructing the microenvironment of lower MNs. Spinal organoids develop dorsoventral patterning and enrich for MNs, enabling studies of spinal-level degeneration (Huang et al., 2024). A recent ALS spinal-chip system, incorporating MNs, vascular cells, and barrier components, distinguished sporadic ALS from controls by revealing early axonal retraction, excitatory–inhibitory imbalance, and impaired barrier integrity (Lall et al., 2025).

At the neuromuscular level, neuromuscular organoids (NMOs) provide a platform for modeling distal degeneration. ALS patient–derived MN–skeletal muscle cocultures exhibit reduced synaptogenesis, impaired neuromuscular transmission, and weakened muscle contraction, paralleling early neuromuscular junction (NMJ) defects in patients (Massih et al., 2023). Self-organizing NMOs with C9ORF72 mutations recapitulate MN degeneration, DPR accumulation, muscle atrophy, and synaptic failure, enabling integrated modeling of neuronal and peripheral pathology (Gao et al., 2024a). Yang et al. (2025) highlight how NMJs, organoid-on-chip motor units, and muscle organoids together form an interconnected ALS modeling framework capable of comparing therapeutic strategies—including antisense oligonucleotides, small molecules, and gene-editing approaches. These multicellular systems are pivotal for evaluating therapies that target complex cell–cell interactions. A notable example is a combination gene therapy targeting SOD1 and neuroinflammation (via galectin-1), which in patient iPSC-derived microglia–motor neuron co-cultures reduced pro-inflammatory cytokines and rescued motor neuron death, demonstrating the utility of such models for validating multitarget strategies (Baird et al., 2024).

Overall, ALS organoid models have evolved from single-cell-type systems to multiscale, cross-tissue frameworks. Cortical organoids capture upper MN pathology, spinal organoids reveal local microenvironment dysfunction, and NMOs model NMJ disruption and distal degeneration. Importantly, NMOs enable phenotypic drug screening at a system level. For instance, acute treatment with the unfolded protein response inhibitor GSK2606414 in C9orf72-ALS NMOs was shown to double glutamate-induced muscle contraction force, providing direct functional evidence of therapeutic efficacy (Gao et al., 2024b). This integrated organoid ecosystem increases the fidelity of *in vitro* ALS modeling and enhances drug screening, mechanism validation, and individualized therapy prediction (Bye et al., 2025).

## 4 Applications of organoids in drug discovery and target validation

With advances in iPSC technology and 3D culture systems, brain organoids have evolved from simple “disease models” into powerful “drug discovery platforms.” They now enable high-throughput compound screening, target identification and validation, individualized drug-response prediction, and neurotoxicity assessment. Recent reviews and methodological studies have systematically evaluated the potential of organoid-based pharmacology, laying the foundation for their integration into standardized preclinical pipelines (Shaikh et al., 2025; Ajongbolo and Langhans, 2025; Giorgi et al., 2024).

### 4.1 High-throughput drug screening and lead compound identification

Conventional 2D cell lines and animal models often fail to balance human relevance, cellular complexity, and high-throughput scalability. Automated organoid platforms and microfluidic technologies now allow parallel screening of hundreds to thousands of compounds. These integrated systems enable automated feeding, real-time imaging, and electrophysiological monitoring, facilitating standardized assessment of compound effects on neural network activity, cell survival, and protein aggregation. Such platforms markedly enhance sensitivity and reproducibility in early-stage drug discovery (Ma et al., 2025; Balusu et al., 2023). Giorgi and colleagues described brain organoids as a “game-changer” for central nervous system (CNS) drug testing due to their ability to capture human-specific phenotypes.

Disease-specific applications have further strengthened this utility. In AD organoids, *β*/*γ*-secretase inhibitors, Tau-targeting compounds, and small molecules modulating APP processing have been systematically evaluated using immunostaining, Enzyme-Linked Immunosorbent Assay (ELISA), and transcriptomic readouts to quantify their effects on Aβ deposition and Tau phosphorylation (Fernandes et al., 2024). In PD research, human midbrain organoids have enabled phenotypic screening of LRRK2 inhibitors, GCase activators, and anti-*α*-syn aggregation compounds, with multidimensional evaluation based on DA neuron viability, mitochondrial function, and neuronal network dynamics (Cui et al., 2024; Qin et al., 2025).

### 4.2 Target discovery and mechanistic validation

Organoids uniquely support the transition from correlation to causation in mechanistic studies. CRISPR–Cas9 editing in iPSCs or organoids allows construction of isogenic pairs carrying or lacking specific pathogenic mutations, enabling direct comparison of phenotypes under identical genetic backgrounds (Ajongbolo and Langhans, 2025; Fernandes et al., 2024). In Down syndrome–derived organoids, manipulation of BACE2 copy number combined with *β*/*γ*-secretase inhibition has provided causal evidence linking APP processing to Aβ accumulation, thereby validating this pathway as a therapeutic target (Papaspyropoulos et al., 2020). In PD midbrain organoids, gene-edited models targeting LRRK2 or GBA1 have been used to dissect the causal relationships among mitochondrial impairment, lysosomal dysfunction, and DA neuron degeneration. When drug rescue experiments reverse these phenotypes, organoids enable unified “target–pathway–phenotype–drug response” verification (Cui et al., 2024; Qin et al., 2025).

### 4.3 Personalized drug response prediction and disease subtyping

Patient-derived iPSC organoids inherently preserve individual genetic backgrounds, making them promising tools for predicting inter-individual variability in drug responses and supporting disease subtype stratification. Emerging translational platforms now offer organoid-based drug screening as contract research services for pharmaceutical development, with applications in therapeutic optimization, neurotoxicity profiling, and response prediction for neurodegenerative disorders (Lall et al., 2025; Lei et al., 2024). The utility of these platforms is significantly enhanced by advanced engineering. For instance, the development of multi-organ chip systems, such as the PEGASO platform which dynamically links intestine, liver, blood–brain barrier, and brain units, has enabled the testing of drug transport and efficacy—for example, assessing the inhibition of acetylcholinesterase activity in brain units following donepezil treatment—in a more physiologically integrated context. Similarly, modular platforms like the “blood–brain barrier (BBB)-in-a-CUBE” integrated with brain cancer models simulate the process of drug penetration across the blood–brain barrier and action on cerebral targets (Koh and Hagiwara, 2024).

In preclinical testing, organoids enable evaluation of candidate therapies across diverse genetic backgrounds to identify potential responder and non-responder subgroups, thereby informing clinical trial design (Giorgi et al., 2024; Gazerani, 2024). Vascularized brain organoids introduce another critical dimension. Notably, exposing neuroimmune organoids with vascular structures to brain extracts from different sporadic Alzheimer’s disease patients has been shown to recapitulate patient-specific pathological features and elicit differential efficacy and vascular inflammatory responses to the anti-Aβ antibody lecanemab, highlighting their potential for personalized efficacy and safety prediction (Ji et al., 2025). Furthermore, engineering midbrain organoids using 3D-printed vasculature-inducing, dispersible scaffolds markedly reduces necrosis and hypoxia. These engineered organoids exhibit more mature neuronal network activity and yield more significant and reliable pharmacological responses, such as quantifiable changes in neural activity upon fentanyl exposure, compared to traditional organoids (Cai et al., 2025).

Within motor neuron diseases, rapid NMJ models derived from iPSCs have shown particular value. Standardized MN–skeletal muscle co-culture systems can reconstruct functional human NMJs within 10–12 days, enabling comparison of drug responsiveness between ALS patient-derived cells and controls. These systems serve as “miniature individualized drug-testing platforms” for precision therapeutics (Gao et al., 2024a).

### 4.4 Neurotoxicity and safety assessment

Neurotoxicity often becomes evident only in late animal studies or early clinical trials. Brain organoids offer a means to detect safety concerns earlier by modeling drug effects in a human-relevant 3D environment. Organoids enable simultaneous evaluation of neuronal survival, synaptic density, network firing patterns, and glial activation, allowing early elimination of compounds that induce synaptic, mitochondrial, or inflammatory toxicity (Shaikh et al., 2025; Ajongbolo and Langhans, 2025; Giorgi et al., 2024; Gazerani, 2024). The development of organoid-on-chip systems and vascularized organoids further permits assessment of BBB permeability, tissue distribution, and pharmacokinetic/pharmacodynamic characteristics under physiologically relevant conditions (Ajongbolo and Langhans, 2025; Gazerani, 2024). Although these platforms are still undergoing standardization and regulatory evaluation, they already demonstrate strong potential as complementary—or even partial replacements—for animal testing.

## 5 Limitations, challenges, and future directions

Despite the rapid rise of brain organoids as powerful platforms for neurodegenerative disease research and drug discovery, substantial limitations remain in terms of physiological maturity, structural complexity, and reproducibility. Most current organoids correspond molecularly and electrophysiologically to fetal or early developmental stages rather than adult or aging human brains. This immaturity restricts their ability to model late-onset pathological processes such as chronic neuroinflammation, age-associated protein aggregation, and mitochondrial decline. Although prolonged culture or optimized induction conditions can partially enhance maturation, they do not fully recapitulate adult myelination, stable neuronal network activity, or fully functional glial populations (Shaikh et al., 2025; Ajongbolo and Langhans, 2025; Giorgi et al., 2024).

A second major limitation is the absence of vasculature and a functional BBB. The lack of vascular systems restricts organoid size, leads to hypoxic or necrotic cores, and prevents accurate evaluation of drug penetration, distribution, and clearance within the CNS. Recent advances in bioengineering—including endothelial-cell integration, biomaterial scaffolds, and microfluidic systems—have enabled partial vascularization and improved nutrient exchange, thereby enhancing their utility for pharmacological studies. Some groups have proposed combining vascularized organoids with organoid-on-chip systems as next-generation platforms for CNS drug testing (Kistemaker et al., 2025; Xu et al., 2024; Giorgi et al., 2024). Additionally, most organoids lack immune components, particularly mature microglia, which limits their ability to model inflammation-driven disease mechanisms or evaluate inflammation-targeting therapeutics. Recent approaches incorporating induced microglial precursors or exogenous immune cells have begun to reconstruct more physiologically relevant neuroimmune interactions, opening opportunities to investigate TREM2, NLRP3, and other inflammatory pathways in AD, PD, and ALS models (Fernandes et al., 2024).

Reproducibility and standardization also remain major concerns. Organoid formation is influenced by donor cell variability, induction protocols, and culture conditions, leading to batch effects that undermine cross-study comparability. Recent studies highlight the urgent need for rigorous quality-control frameworks to monitor parameters such as cellular composition, maturation state, transcriptional trajectories, metabolic profiles, and electrophysiological properties (Yang et al., 2024; Lee et al., 2024). Automated culture platforms and imaging/omics-based quality-control pipelines are expected to enhance scalability and consistency in future organoid workflows (Shaikh et al., 2025; Ajongbolo and Langhans, 2025; Giorgi et al., 2024).

At the structural and functional levels, organoids still struggle to model long-range neuronal connectivity and higher-order circuit dynamics. To address this, assembloid technologies—fusing multiple region-specific organoids (e.g., cortex–midbrain or thalamus–cortex)—have emerged as promising systems for reconstructing interregional pathways and modeling complex phenomena such as Tau or *α*-syn spread, axonal guidance defects, and circuit desynchronization (Xiang et al., 2017). As these methods mature, assembloids may enable a transition from “cell-level modeling” to true “systems-level modeling,” potentially becoming indispensable tools for developing circuit-targeting therapies (Ting et al., 2025).

Future development will require not only improved maturation, vascularization, and immune integration but also the adoption of a “multi-modal readout + multi-omics integration + AI-driven analytics” paradigm. Large-scale datasets generated from single-cell omics, proteomics, electrophysiology, live imaging, and metabolic profiling can greatly advance our understanding of disease drivers, drug-response heterogeneity, and therapeutic target identification. AI—including deep learning and network pharmacology models—is increasingly incorporated into organoid research to predict drug effects, stratify disease subtypes, and design individualized therapeutic strategies. With the integration of automated production, high-content imaging, microfluidic systems, and computational modeling, organoids are gradually evolving into “human-relevant” evaluation platforms for early-stage CNS drug development (Gao et al., 2024b; Shaikh et al., 2025).

**Figure 2:**
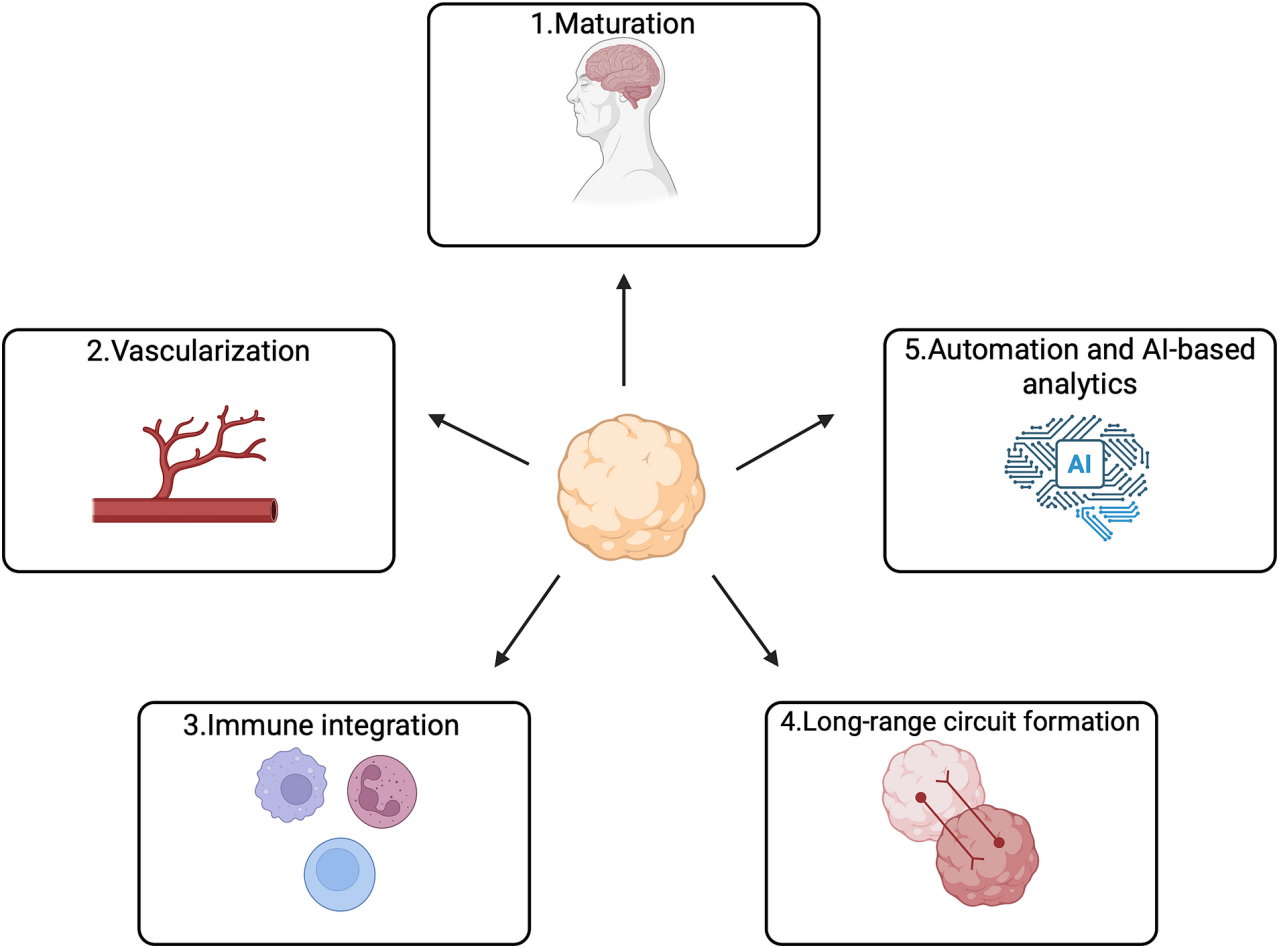
The future development directions of organoid technology. Expected advancements in vascularization, immune integration, long-distance circuit formation, automation, and AI-driven analytics are anticipated to allow organoids to fulfill the requirements for human-relevant preclinical screening.

Overall, organoid technology is undergoing a critical transition from basic research models to engineered, standardized platforms suitable for translational pharmacology and toxicology. Although challenges remain in maturity, microenvironmental fidelity, functional complexity, and reproducibility, rapid progress in vascularization, immune incorporation, long-range circuit formation, automation, and AI-based analytics is expected to enable organoids to meet the demands of human-relevant preclinical screening. As such, future organoid systems may serve not as replacements for animal models but as essential human-derived bridges linking molecular/cellular studies to clinical research—providing increasingly valuable tools for drug discovery, target validation, and personalized therapeutic development in neurodegenerative diseases (Figure 2).

## 6 Conclusion

Neurodegenerative diseases such as AD, PD, and ALS present complex pathogenic mechanisms and remain largely incurable, posing major challenges to neuroscience and clinical medicine. Although traditional animal models and 2D culture systems have contributed substantially to mechanistic insights over past decades, species differences and the absence of a 3D human neural microenvironment limit their ability to faithfully recapitulate disease phenotypes and predict therapeutic efficacy—factors that have contributed to repeated failures of candidate drugs in clinical translation. iPSC-derived brain organoids offer a promising solution to these longstanding gaps. Current evidence demonstrates that organoids can reconstruct key pathological events—including Aβ/Tau pathology, DA neuron vulnerability, *α*-syn aggregation, TDP-43 mislocalization, and NMJ disruption—within a human 3D context, and have already been integrated into drug screening, drug-response profiling, and target validation pipelines. The combination of organoids with CRISPR–Cas9-based genome engineering and multi-omics technologies further enables causal mechanistic interrogation and individualized drug-response prediction.

Nevertheless, organoid systems remain limited by their immature developmental states, lack of vasculature and immune microenvironments, batch variability, and incomplete standardization. These constraints prevent organoids from fully replacing animal models in the near term. Future advances will likely depend on progress in vascularization, immune incorporation, cross-regional assembloid construction, rigorous quality control, automated culture systems, and AI-driven multimodal data integration.

Taken together, brain organoids should not be viewed merely as substitutes for animal models but rather as transformative human-derived platforms that bridge molecular and cellular studies with translational and clinical research. As organoid technologies continue to mature and increasingly interface with engineering and computational biology, they are poised to become indispensable components of CNS drug-development pipelines, offering new opportunities for mechanistic discovery, therapeutic innovation, and precision medicine in neurodegenerative diseases.
